# Real-Time Reconstruction of the Temperature Field of NSRT’s Back-Up Structure Based on Improved RIME-XGBoost

**DOI:** 10.3390/s25247410

**Published:** 2025-12-05

**Authors:** Shi-Jiao Zhang, Qian Xu, Hui Wang, Fei Xue, Fei-Long He, Xiao-Man Cao

**Affiliations:** 1 Xinjiang Astronomical Observatory, Chinese Academy of Sciences, Urumqi 830011, China; 2 University of Chinese Academy of Sciences, Beijing 100049, China; 3 State Key Laboratory of Radio Astronomy and Technology, Beijing 100101, China; 4 Xinjiang Key Laboratory of Radio Astrophysics, Urumqi 830011, China

**Keywords:** radio telescope, back-up structure, environmental sensing, temperature field, thermal prediction model, machine learning, eXtreme Gradient Boosting (XGBoost), meta-heuristic algorithms, RIME optimization algorithm

## Abstract

Obtaining an antenna’s back-up structure (BUS) temperature field is an essential prerequisite for analyzing its thermal deformation. Thermodynamic simulation can obtain the structure’s thermal distribution, but it has low computational accuracy. There is a problem with cumbersome wiring and difficult maintenance of the temperature measurement system. This study developed an improved RIME-XGBoost model to realize the temperature prediction of the BUS of the Nanshan 26-m Radio Telescope (NSRT). The proposed model successfully predicts the NSRT’s BUS temperature distribution based solely on environmental sensing (ambient temperature, angle of solar radiation, antenna’s orientation, etc.). The relative prediction accuracy between the predicted and actual BUS temperature is 97.15%, and the predictive error is less than 0.897 K (root mean square error, RMSE). This research result provides an alternative method for the real-time reconstruction of the structure’s thermal distribution in large-aperture radio telescopes.

## 1. Introduction

With the increase in the aperture of radio telescopes and the improvement in observation frequency bands, there are higher requirements for the surface accuracy and pointing error of radio telescopes. However, large-aperture radio telescopes are susceptible to solar radiation, wind gusts, ambient temperature, relative humidity, and other environmental factors, which can degrade antenna performance. Among these factors, the non-uniform temperature field acting on the antenna structure is a critical factor in the decrease in surface accuracy and pointing accuracy. Greve et al. [1] found through finite element analysis of the IRAM (Institute of Radioastronomie Millimétrique) 30-m telescope that a 0.1 K change in the antenna structure temperature difference would result in a 0.005 mm surface error. Li et al. [2] conducted a simulation analysis on the effect of sunshine temperature deformation on a 65-m radio telescope’s surface accuracy, and the result shows that a 1 K temperature difference changes the surface accuracy component by about 0.09 mm. As reported by Ukita et al. [3], measurements performed on the 10-m ASTE (Atacama Submillimeter Telescope Experiment) telescope revealed that the pointing error induced by temperature difference is approximately 1.5 arcseconds per degree. Therefore, achieving high-precision and real-time acquisition of temperature changes in antenna structures is a necessary prerequisite for improving the performance of radio telescopes.

A series of relevant studies have been carried out on the thermal analysis of the radio telescope structure. Attoli et al. [4] analyzed the Sardinia Radio Telescope using the finite element method to determine the pointing error generated by each simulated thermal scenario. Nikolic et al. [5] used phase-retrieval holography measurement on the GBT (Green Bank Telescope) to demonstrate that thermal deformation usually exceeds gravitational deformation during daytime. Wei et al. [6] analyzed the temperature distribution of the primary reflector of a 70-m radio telescope using the finite element method (FEM). Meanwhile, through on-site measurements with an optical camera and a thermal imaging camera, they found that the temperature distribution of the primary reflector under solar radiation is very uneven, and the maximum of the root mean square temperature is 12.3 K. In summary, the main methods for thermal acquisition of antenna structures include thermodynamic simulation and on-site measurement. The BUS temperature field is challenging to obtain accurately through thermodynamic simulation. The temperature sensors can obtain the structure temperature, but problems include electromagnetic interference, difficult maintenance, complicated sensor wiring, and easy wear and tear. Finally, it is challenging to realize the long-term, stable, real-time acquisition of the BUS temperature. So far, there is no efficient and feasible method to realize the real-time acquisition of the thermal distribution.

In order to achieve real-time and high-precision acquisition of the BUS temperature information, we attempt to use surrogate models or neural networks to solve this problem. Indeed, researchers have attempted to realize real-time structure temperature prediction without temperature sensors by leveraging machine learning or neural network models based on long-term historical temperature data. For instance, Yan et al. [7] proposed a new multi-data-driven model based on reinforcement learning and deep learning for high-precision prediction of locomotive axle temperature. Hu et al. [8] proposed a multi-scale convolutional neural network to predict the final cooling temperature, with a prediction error of approximately ± 10% of the actual temperature. Li et al. [9] proposed a hybrid model that combines a physical model with a long short-term memory (LSTM) network for predicting temperature during spacecraft thermal testing. The temperature prediction accuracy of this method is MAE (mean absolute error) = 17.41 K, $R^{2}$ (the coefficient of determination) = 0.9988. However, there are no experimental cases yet attempting to use neural network models to achieve thermal acquisition of radio telescope structures.

In this study, we propose a model for the real-time reconstruction of the structure’s temperature field. Building the prediction model using a data-driven approach can replace complex or unknown mathematical equations. The working environment of the NSRT is taken as the input, which contains the NSRT’s orientation, ambient temperature, and relative humidity, the angle of solar radiation, etc. The measured data for 8 months is used as the model training dataset, and then the model parameters are optimized to obtain the best prediction model. Finally, the model realizes the real-time prediction of the radio telescope’s BUS temperature based on environment sensing. This method analyzes the weights of the factors affecting the BUS temperature distribution, realizing the prediction of the BUS temperature distribution solely based on the environmental sensing, without relying on the real-time temperature sensors.

## 2. Data Collection and Feature Analysis

### 2.1. Data Collection

The data collection system consists of a structure temperature measurement system and an environment monitoring system. The data were collected from 15 November 2021 to 15 May 2022 [10]. The time interval of the data is 10 s, which includes ambient temperature, relative humidity, barometric pressure, wind direction, wind speed, the antenna’s azimuth and elevation, solar altitude and azimuth, and the temperature of 45 locations on the NSRT’s BUS.

**Figure 1:**
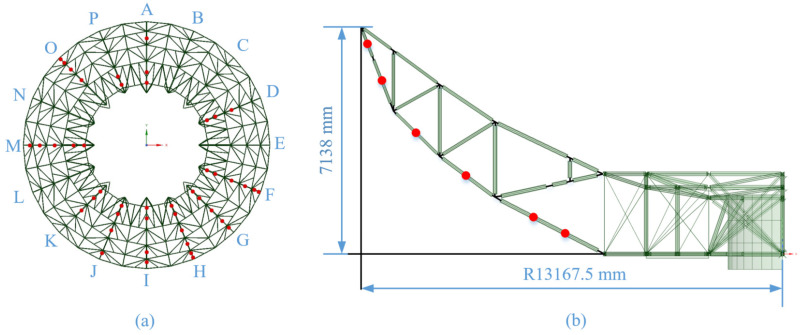
Diagram of temperature sensors’ deployment position. (**a**) Top view. (**b**) Cross-sectional view.

In total, 45 FBG (Fiber Bragg Grating) temperature sensors were installed on the NSRT’s BUS [11,12]. Fiber-Optic Grating Sensors have been widely applied in structure health monitoring scenarios due to their advantages, such as immunity to electromagnetic interference, small size, light weight, durability, and high bandwidth, which allow a significant number of sensors to operate in the same system [13,14]. The specific installation positions of the temperature sensors are shown in Figure 1, in which Figure 1a is the top view of the BUS and A-P indicates the 16 measurement channels of the 16 main radial beams. Figure 1b is the front view of a single radial beam M, and the dots are the mounting positions for these sensors. It can be seen that all of the sensors are deployed at equidistant points on the lower chord beams.

**Figure 2:**
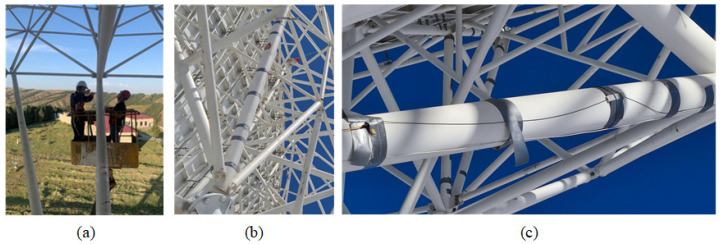
Diagram of temperature sensor field installation. (**a**) Manned crane for laying sensors. (**b**) Sensor layout global diagram. (**c**) Sensor layout detail diagram.

The field installation of temperature sensors is shown in Figure 2. Figure 2a shows the manned spider crane positioned at the lower chord beam of the BUS to install the temperature sensor. The global diagram of the sensor mounting positions on the BUS is shown in Figure 2b. The optical lines and temperature sensors are fixed with moisture-proof, waterproof, heat-insulating, and wear-resistant grey PTFE (polytetrafluoroethylene) film tape. The local detail sensor mounting diagram is shown in Figure 2c.

**Figure 3:**
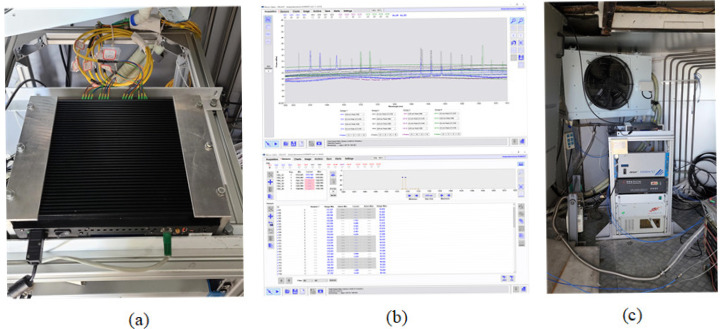
Diagram of the BUS temperature data collecting equipment. (**a**) Temperature sensor collection device. (**b**) Software interface of the BUS temperature. (**c**) Global view of the temperature collection device.

The temperature acquisition equipment in the NSRT high-frequency cabin is shown in Figure 3. The fiber-optic lines transmit the Bragg wavelength information collected by each temperature sensor to the high-frequency cabin, as shown in Figure 3a. The temperature value interface of all temperature sensors was obtained from 16 optical fibers processed by the FBG demodulator, as shown in Figure 3b. The data processing equipment of the FBG temperature measurement system is shown in Figure 3c.

**Figure 4:**
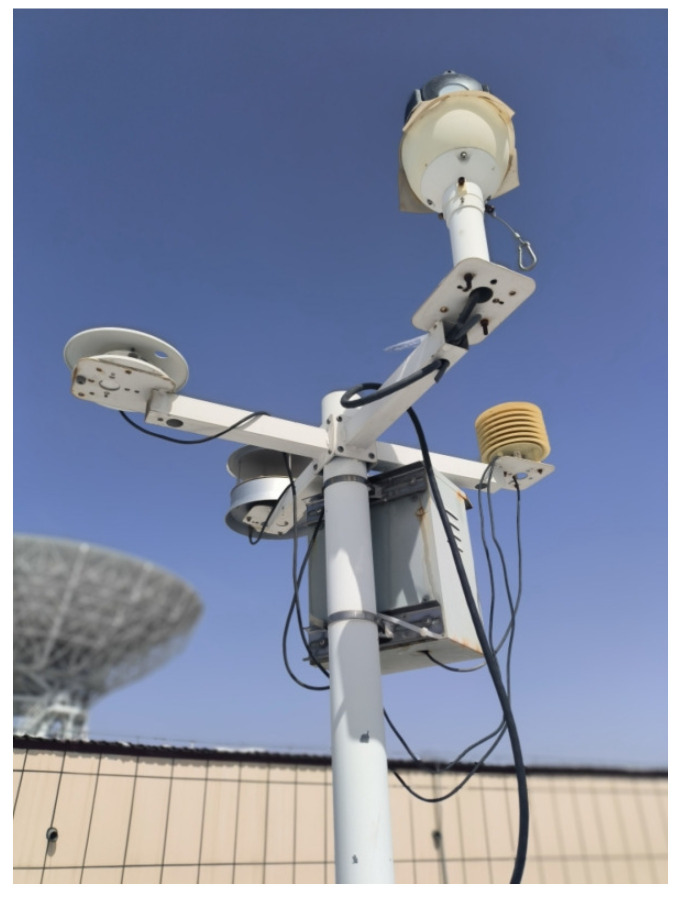
Diagram of multi-element meteorological station.

The environmental data collection equipment is a multi-functional integrated weather station built near the NSRT, which can detect wind direction, wind speed, ambient temperature, relative humidity, barometric pressure, and other meteorological elements. The overall weather station consists of three parts, the meteorological sensors, the data logger, and the data acquisition system, of which the meteorological sensors on the pole are shown in Figure 4.

The solar altitude and elevation are calculated from the NSRT geographic coordinates and the current time using Python 3.9 code. The NSRT’s azimuth and elevation are collected from the NSRT real-time operating log and will not be repeated here.

### 2.2. Data Analysis

**Figure 5:**
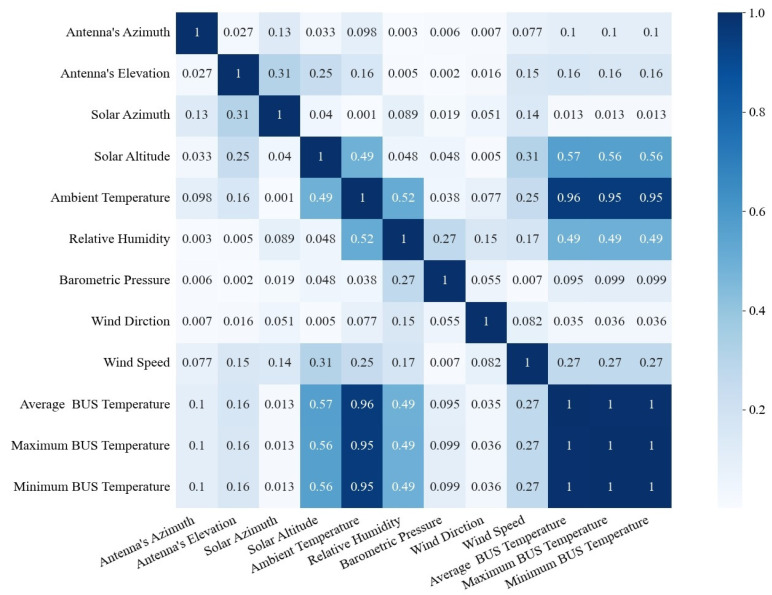
Heat map of the correlation between the BUS temperature and the ambient factors.

After collecting the BUS temperature and the working condition data, the feature analysis of each data variable is carried out to explore their correlation with the BUS temperature. The heat map of the Pearson coefficient matrix is used to measure the correlation between the features, as shown in Figure 5 [15]. Only the mean, maximum, and minimum BUS temperature values are counted to simplify the expression of the BUS temperature data.

**Figure 6:**
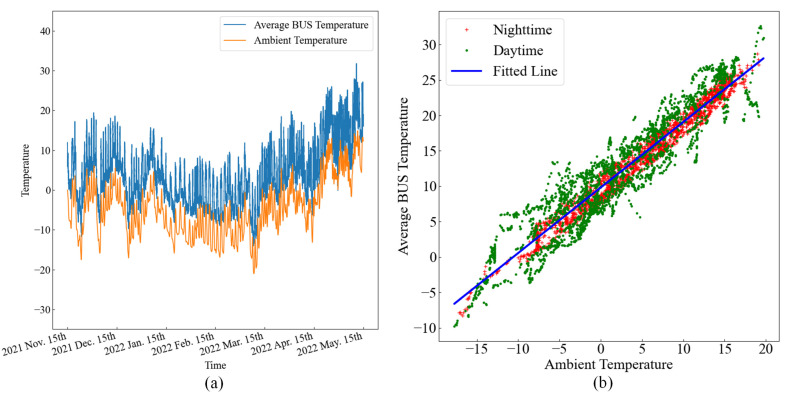
Diagram of the BUS temperature and ambient temperature. (**a**) Time-varying diagram of the BUS temperature and ambient temperature. (**b**) Relational graph of the BUS temperature and ambient temperature between day and night.

As can be seen from Figure 5, the BUS temperature is strongly related to the ambient temperature. The solar altitude and the relative humidity also have a certain correlation with the BUS temperature. The following analyzes the ambient temperature, relative humidity, and solar altitude in turn. Figure 6a shows the variation in the average BUS temperature and the ambient temperature over time, and Figure 6b shows the relationship between the BUS temperature and the ambient temperature during the day and night. From Figure 6, it is concluded that the overall BUS average temperature is highly correlated with the ambient temperature in a linear way [16], especially at night, and the goodness of fit of the $R^{2}$ is 0.92481.

Table 1 shows the statistical result of the BUS temperature. From the data in Table 1, the maximum of the 45 points on the BUS is 28.28 K higher than the ambient temperature, and the average temperature of the whole structure is 12.29 K higher than the ambient temperature, which shows that the temperature field of the BUS is greatly affected by the change in the ambient temperature. In addition, the BUS temperature strongly correlates with the ambient temperature, and its large temperature drift mostly occurs at noon in summer.

As for the effect of the ambient humidity on the BUS temperature, there is no apparent correlation. The effect of the solar altitude on the BUS temperature is related to the combination of the NSRT’s orientation (heating angle) and the rotation speed, making it challenging to find the pattern using mathematical statistics. As for the effect of wind, barometric pressure, and other factors on the BUS, we will not carry out their feature analysis here because the correlation between them is weak and the features are not prominent.

## 3. Development of the Model

### 3.1. The XGBoost Model

eXtreme Gradient Boosting (XGBoost) is a C++ implementation of the Gradient Boosting Machine algorithm by Tianqi Chen at the University of Washington [17], and it is highly suitable for obtaining structure temperature fields [18,19,20]. XGBoost makes a second-order Taylor expansion of the loss function and adds a regularization term to the loss function to control the complexity of the model and avoid overfitting. Meanwhile, XGBoost utilizes the central processing unit’s multithreaded parallel operation to improve computational efficiency greatly. The objective function of the XGBoost model can be written as follows:

(1) $$Obj=\sum_{i=1}^{N}L\left(y_{i},{\hat{y}}_{i}\right)+\sum_{j=1}^{t}\Omega \left(g_{j}\right),\Omega \left(g_{j}\right)=\mathrm{YM}+\frac{1}{2}\lambda \sum_{j=1}^{M}\omega_{j}^{2}$$

where Obj denotes the objective function, $L(y_{i},{\hat{y}}_{i})$ is the loss function, $\Omega (g_{j})$ is the regularization term, also known as the complexity of the regression tree, M is the number of leaf nodes in the decision tree, $\omega_{j}$ denotes the node value, and Y and λ are hyperparameters to control the penalty strength.

### 3.2. The RIME Algorithm

The rime optimization algorithm (RIME) is a new heuristic optimization algorithm based on the physical phenomenon of rime-ice in nature [21]. The RIME algorithm implements exploration and exploitation behaviors in optimization by simulating the soft-rime and hard-rime growth process of rime-ice and constructing a soft-rime search strategy and a hard-rime puncture mechanism. In the initialization phase, the RIME algorithm initializes the whole rime-population R. The rime-population consists of *p* rime-agents $S_{p}$, and each rime-agent consists of *q* rime-particles $X_{pq}$. Thus, the rime-population R can be directly represented by the rime-particles X, as shown in Equation (2).

(2) $$R=\left[\begin{array}{c} S_{1}\\ S_{2}\\ \vdots \\ S_{p} \end{array}\right];S_{p}=\left[\begin{array}{cccc} X_{p1} & X_{p2} & \cdots & X_{pq} \end{array}\right];R=\left[\begin{array}{cccc} X_{11} & X_{12} & \cdots & X_{1q}\\ X_{21} & X_{22} & \cdots & X_{2q}\\ \vdots & \vdots & \ddots & \vdots \\ X_{p1} & X_{p2} & \cdots & X_{pq} \end{array}\right]$$

From Formula (2), it can be seen that to initialize rime-population R, one needs to initialize its smallest unit rime-particles X. X is a candidate solution, and in this experiment, rime-particles X represent a set of hyperparameters of the XGboost model. If there are d hyperparameters, then X can be expressed as $X=\left[\begin{array}{c} x_{1},x_{2},x_{3},\cdots ,x_{d} \end{array}\right]$, where $x_{d}$ represents the *d*th hyperparameter, and the process of the hyperparameter $x_{d}$ is shown in Formula (3).

(3) $$X_{d}={Lower}_{d}+rand\left(0,1\right)\left({Upper}_{d}-{Lower}_{d}\right)$$

where ${Lower}_{d}$ is the lower bound of the *d*th hyperparameter. ${Upper}_{d}$ is the upper bound of the *d*th hyperparameter. rand(0,1) is a random number within the range of 0 to 1.

Furthermore, $G\left(S_{p}\right)$ is used to denote the growth state of each rime-agent, i.e., the fitness value of the agent in the meta-heuristic algorithm; the expression is shown Equation (4).

(4) $$G\left(S_{p}\right)=\left[\begin{array}{cccc} f([X_{11} & X_{12} & \cdots & X_{1q}])\\ f([X_{21} & X_{22} & \cdots & X_{2q}])\\ \vdots & \vdots & \ddots & \vdots \\ f([X_{p1} & X_{p2} & \cdots & X_{pq}]) \end{array}\right]$$

where *f* is the adaptation degree of each rime-particle.

Each rime-particle $X_{pq}$ will move according to a certain law before condensation, and the free particles will move to the soft-rime neighborhood to condense, but cannot condense when exceeding a certain range. Aiming at the motion properties of the rime-particles, the RIME algorithm proposes the soft-rime search strategy, as shown in Equation (5).

(5) $$R_{pq}^{\mathrm{new}}=R_{\mathrm{best},q}+r_{1}\cos\theta \beta \left(h\left(Ub_{pq}-Lb_{pq}\right)+Lb_{pq}\right),r_{2}<E$$

where $\theta =\pi k/\left(10K\right),\beta =1-\left[\left(wk\right)/K\right]/w,E=\sqrt{\left(k/K\right)}$, $R_{pq}^{\mathrm{new}}$ is the new position of the updated particle, and *p* and *q* denote the *q*-th particle of the *p*-th rime-agent. $R_{\mathrm{best},q}$ is the *q*-th particle of the best rime-agent in the rime-population R.

The increase in the soft-rime area enables the algorithm to cover the full space search quickly. Using the hard-rime puncture phenomenon to achieve dimensional crossover interchange between ordinary and optimal agents improves the ability to jump out of the local optimum. The formula for replacement between particles is shown in Equation (6).

(6) $$R_{pq}^{\mathrm{new}}=R_{\mathrm{best},q},r_{3}<F^{\mathrm{normr}}\left(S_{p}\right)$$

where $F^{\mathrm{normr}}\left(S_{p}\right)$ denotes the normalized value of the current agent fitness value, indicating the chance of the *p*-th rime-agent being selected, and $r_{3}$ is a random number within (−1,1).

After the Soft Frost Search (exploration) and Hard Frost Perforation (exploitation) are completed, a forward greedy selection mechanism should be used to filter the updated solutions, thereby indirectly ensuring the balance effect between the two. The specific approach is to compare the updated fitness value of a rime-agent S with the fitness value of a rime-agent S before the update. If the updated fitness value is better than the value before the update, the individual (rime-agent S) will be replaced. On one hand, this mechanism actively replaces individuals, enabling the rime-population R to continuously have good individuals, thereby improving the quality of the global solution. On the other hand, as the position of the rime-agents S of the rime-population R changes significantly in each iteration, there will inevitably be rime-agents S that perform worse than the rime-population R before the update. Therefore, this operation can be used to ensure that the rime-population R evolves in a more optimal direction in each iteration. The specific determination formula is shown in Equation (7):

(7) $$if\;F\left(R_{p}^{\mathrm{new}}\right)<F\left(R_{p}\right):F\left(R_{p}\right)=F\left(R_{p}^{\mathrm{new}}\right),R_{p}=R_{p}^{\mathrm{new}}$$

where $F(R_{p}^{\mathrm{new}})$ is the fitness value of the new individual, while $F(R_{p})$ is the fitness value before the update.

### 3.3. Algorithm Improvement

In order to overcome the shortcomings of the RIME algorithm, such as poor initial sensitivity and easy overfitting, the RIME cluster initialization is optimized using chaotic mapping. In addition, the search agent uses refracted opposition-based learning to obtain the opposite solution in the solution space so as to enhance the global search ability and increase the possibility of the algorithm finding a better solution.

#### 3.3.1. Sine Chaotic Map

Sensitivity to initial conditions and control parameters are the main features of chaotic maps. Generally, simple linear or non-linear equations are used to create chaotic behaviors, and Table 2 shows several classical forms of chaotic mapping.

Different chaotic maps have their own characteristics, which are not repeated here. It should be emphasized that a sine map is simple and efficient, but also has the disadvantage of an uneven probability density distribution. However, the soft-rime search strategy of the RIME algorithm can make up for the shortcomings of the sine map. Therefore, the sine map is used to optimize the RIME cluster initialization instead of the random initialization. This increases the diversity of the rime-population and avoids falling into local optimization during the search process of the RIME algorithm. The improved algorithm for rime-population R initialization is actually an improvement on its basic unit rime-particles X. The specific improved process is shown in Formula (8).

(8) $$X_{d}={Lower}_{d}+sin\left(\pi \cdot rand\left(0,1\right)\right)\left({Upper}_{d}-{Lower}_{d}\right)$$

where ${Lower}_{d}$ is the lower bound of the *d*th hyperparameter. ${Upper}_{d}$ is the upper bound of the *d*th hyperparameter. rand(0,1) is a random number within the range of 0 to 1. sin() is the sine map function.

#### 3.3.2. Refractive Opposition-Based Learning

The refractive opposition-based learning strategy (ROBL) is a modification of the opposition-based learning strategy [22], which enhances the optimization algorithm’s accuracy by combining the principle of light refraction with the OBL’s advantage of calculating the opposite solution to expand the population search space.

**Figure 7:**
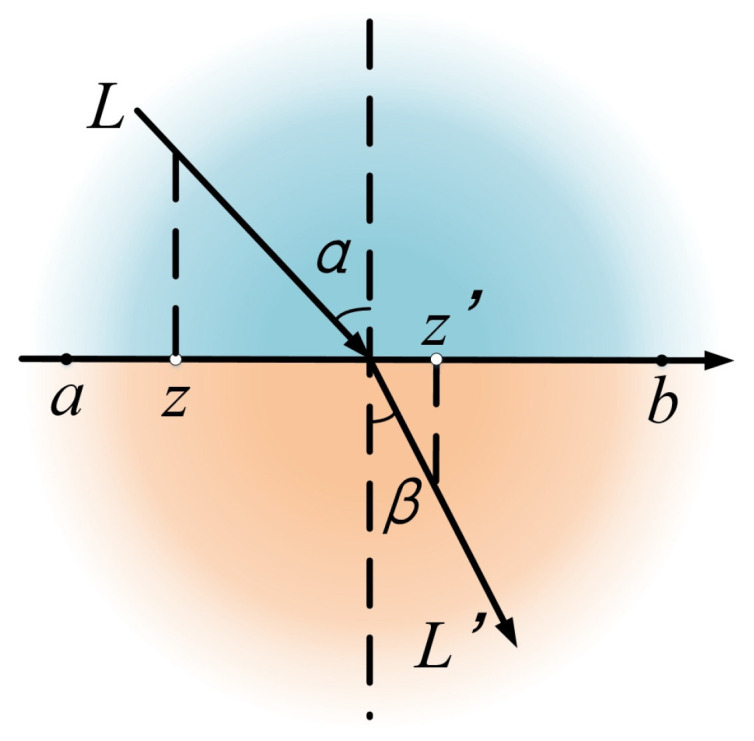
Schematic diagram of the light refraction.

As shown in Figure 7, the algorithmic core of the refractive opposition-based learning strategy draws on the principle of light refraction. α is the angle of incidence, β is the angle of refraction, and *z* and $z^{\prime }$ are two points within the coordinate axes [a,b]. *L* is the length of the incident ray. $L^{\prime }$ is the length of the refracted ray. The sinusoidal expressions for the angles of incidence and refraction are shown in Equation (9):

(9) $$z^{\prime }=\frac{a+b}{2}+\frac{a+b}{2\varepsilon n}-\frac{z}{\varepsilon n},\varepsilon =\frac{sin(\alpha )}{sin(\beta )}=\frac{\left(\left(a+b\right)/2-z\right)/L^{\prime }}{(z^{\prime }-\left(a+b\right)/2)/L}$$

where $z^{\prime }$ denotes the final solution.

In this algorithm, when ε is less than 1, the search agent maps to other regions to increase the possibility of finding the global optimal region. When ε is greater than 1, the search agent obtains the inverse solution in the local region. Eventually, this way iteratively converges to the optimal solution. The ROBL strategy is applied to the RIME algorithm to improve the balance between the Soft Frost Search (exploration) and Hard Frost Perforation (exploitation). Before selecting the optimal solution for this iteration through the forward greedy selection mechanism, we first perform ROBL optimization on the updated individual (rime-agents S) fitness, as shown in Equation (10):

(10) $$F\left(R_{p}^{\mathrm{ROBL}}\right)=\varepsilon F\left(R_{p}^{\mathrm{new}}\right),\varepsilon =1+\frac{k}{K}\left(\frac{F(R_{p})}{F(R_{p}^{\mathrm{new}})}-1\right)$$

where *k* is the current iteration number, while *K* is the maximum number of iterations. $F(R_{p})$ is the individual fitness value before this iteration update. $F(R_{p}^{\mathrm{new}})$ is the individual fitness value of this update. $F(R_{p}^{\mathrm{ROBL}})$ is the fitness value after applying the RBOL strategy.

Furthermore, the forward greedy selection mechanism in the RIME algorithm should also be modified accordingly. The modified formula is shown in Equation (11):

(11) $$if\;F\left(R_{p}^{\mathrm{ROBL}}\right)<F\left(R_{p}\right):F\left(R_{p}\right)=F\left(R_{p}^{\mathrm{ROBL}}\right),R_{p}=R_{p}^{\mathrm{new}}$$

From Formula (10), it can be concluded that at the beginning of the iteration, the fitness of $F(R_{p}^{\mathrm{ROBL}})$ is very close to that of $F(R_{p}^{\mathrm{new}})$. This is set to accelerate the iteration process and make the rime-population R approach the optimal solution more quickly. As the iteration progresses to a later stage, the rime-population’s iteration speed is controlled by ε, and ultimately a better solution is obtained.

### 3.4. Modeling the Improved RIME-XGBoost

**Figure 8:**
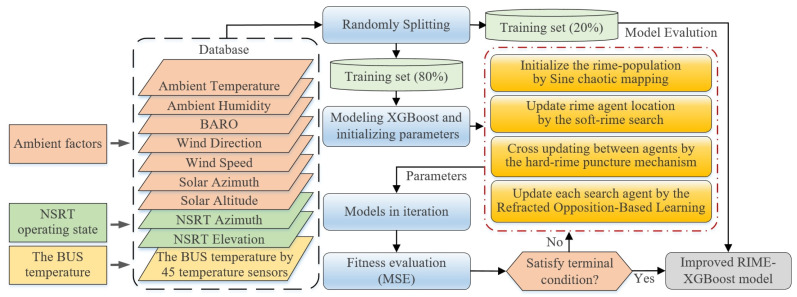
Flowchart of the improved RIME-XGBoost model.

This paper proposes an improved RIME-XGBoost model that uses a multi-strategy optimized algorithm. It integrates the processes of dataset partitioning, improved RIME-XGBoost model training, hyper-parameter optimization, and evaluation index calculation, as shown in Figure 8. The core pseudo-code of the improved RIME algorithm is demonstrated in Algorithm 1.
**Algorithm 1** Pseudo-code of improved RIME algorithm.Initialize the rime-population R using Equation (8) //Improved initialization based on **sine map**Get the current optimal agent and optimal fitness**while** k≤K **do** //*k* is the current iteration number, while *K* is the maximum number of iterations.    Calculate the adherence coeffient of the rime agents $E=\sqrt{\left(k/K\right)}$    **if** $r_{2}<E$ **then**        Update rime agent location by the soft-rime search strategy, referring to Formula (5)    **end if**    **if** $r_{3}<F^{\mathrm{normr}}\left(S_{p}\right)$**then** //$F^{\mathrm{normr}}\left(S_{p}\right)$ is the normalized value of the current agent fitness value        Cross updating between agents by the hard-rime puncture mechanism, referring to Formula (6)    **end if**    Optimize the updated population fitness values using the **ROBL strategy**, referring to Formula (10)    **if** $F\left(R_{p}^{\mathrm{ROBL}}\right)<F\left(R_{p}\right)$ **then**        Select the optimal solution and replace the suboptimal solution using the positive greedy selection mechanism, referring to Formula (11)    **end if**    k=k+1**end while**

The major steps are listed below: (1) The model uses three major factors as inputs: ambient factors, NSRT operating state, and the BUS temperature. Then we divide the data into training (80%) and testing (20%) datasets. (2) For modeling XGBoost, parameters such as the number of trees, the learning rate, and the maximum depth of the tree are selected as the objective optimization parameters. (3) The parameters selected for the model are optimize. Firstly, the RIME population is initialized by the sine map. Then the RIME algorithm is used to update the population. Eventually, the ROBL strategy is used to iteratively converge to the optimal solution. (4) The model’s fitness is evaluated according to the error values, i.e., MSE. If the model errors do not satisfy the stop condition, we continue to execute step (3) iteratively. The specific expression of the mean squared error (MSE) as the stop condition is entered as follows:

(12) $$\mathrm{MSE}=\frac{\sum_{i=1}^{N}\left(y_{i}-{\hat{y}}_{i}\right)}{N}$$

In Equation (12), $y_{i}$ is the actual value, ${\hat{y}}_{i}$ denotes the predicted value, and N is the number of samples. Generally speaking, a smaller MSE indicates better model performance. (5) The optimal parameters of RIME-XGBoost are output to obtain the final model. Meanwhile, testing datasets are used to verify the model’s prediction accuracy. Finally, the final model is saved for direct use later on.

### 3.5. Statistical Criteria for Model Assessment

In order to evaluate the dependability of the model effectively in this research, several indicators such as the root mean square error (RMSE), the mean absolute error (MAE), and the coefficient of determination ($R^{2}$) are used to represent the relationship between the actual BUS temperature value and the predicted value. Below, each of these evaluation indices is explained individually.

The root mean square error (RMSE) has the same dimension and unit as the target variable (i.e., its unit is degrees Celsius), facilitating intuitive analysis of error conditions. However, due to the squaring operation involved, it tends to amplify the impact of outliers. The larger the RMSE is, the greater the deviation between the predicted value and the actual value will be. Its calculation formula is shown in Formula (13):

(13) $$\mathrm{RMSE}=\sqrt{\frac{\sum_{i=1}^{N}{\left({\hat{y}}_{i}-y_{i}\right)}^{2}}{N}}$$

where $y_{i}$ is the actual value, ${\hat{y}}_{i}$ is the predicted value, $\bar{y}$ is the average value, and N is the number of samples.

Compared to RMSE, the mean absolute error (MAE) is robust to outliers and not significantly affected by extreme values, allowing it to truly reflect the average deviation level of most samples. It also has the same dimension and unit as the target variable (i.e., its unit is degrees Celsius). Its calculation formula is shown in Formula (14):

(14) $$\mathrm{MAE}=\frac{\sum_{i=1}^{N}{\left|y_{i}-{\hat{y}}_{i}\right|}^{2}}{N}$$

The coefficient of determination ($R^{2}$) measures the extent to which the model explains the variance in $y_{i}$. It is commonly used for comparisons among different models, and its value ranges from 0 to 1. The closer the value is to 1, the better the model’s fitting performance. Its calculation formula is shown in Formula (15):

(15) $$R^{2}=1-\frac{\sum_{i=1}^{N}{\left(y_{i}-{\hat{y}}_{i}\right)}^{2}}{\sum_{i=1}^{N}{\left(y_{i}-\bar{y}\right)}^{2}}$$

## 4. Model Validation and Results Analysis

### 4.1. Experimental Environment Configuration

The experiments results of this paper were processed in Python 3.9. The software environment was a Windows 10 64-bit operating system. The CPU was an Intel Core i9-9900K processor (Intel, Santa Clara, CA, USA). The memory was a Samsung DDR4 3200 MHz 32 GB RAM (Samsung, Suwon, Republic of Korea). The GPU was an NVIDIA GeForce RTX 3080 10 GB (Nvidia, Santa Clara, CA, USA).

### 4.2. Comparative Analysis with Common Models

In the context of model prediction, the model frameworks adopted for similar application scenarios should generally be quite similar. Indeed, some researchers have employed neural networks, and scholars have attempted to realize real-time temperature prediction of structures without temperature sensors by leveraging machine learning or neural network models based on long-term historical temperature data [7,8,9,19]. Although these models are all used for structure thermal prediction scenarios, the specific prediction objects differ significantly, leading to distinct differences in the training datasets of the models. Consequently, the scene migration adaptability of different models varies. Therefore, to comprehensively analyze the performance of the proposed model in this paper, a comparative analysis will be conducted between GRU (Gated Recurrent Unit), CNN (Convolutional Neural Network), LSTM (Long Short-term Memory), Transformer-BiLSTM, and the proposed model. Table 3 shows the model parameters required for each model. The training and testing results are presented in Table 4.

Combined with the performance results of each model in Table 4, the prediction accuracy and fitting ability are analyzed. The improved RIME-XGBoost model demonstrates the highest prediction accuracy and fitting capability across all evaluated metrics. It achieves an extremely low MAE of 0.331 K (train) and 0.621 K (test), an RMSE of 0.369 K (train) and 0.897 K (test), and an $R^{2}$ of 0.999 K (train) and 0.996 K (test) in the training and test stages, respectively. This indicates that the improved ensemble learning framework (XGBoost) excels in capturing complex patterns within the data. Following this, the Transformer-BiLSTM model exhibits remarkable performance, with MAE values of 1.08 K (train) and 1.166 K (test), RMSE values of 1.458 K (train) and 1.543 K (test), and $R^{2}$ values of 0.988 (train) and 0.984 (test). The integration of Transformer and BiLSTM enables effective capture of long-range dependencies and sequential features, leading to superior accuracy compared to traditional deep learning models. In contrast, traditional deep learning models such as GRU, CNN, and LSTM exhibit moderate performance. Among them, CNN has a relatively better $R^{2}$ (0.962 for training and 0.959 for testing), while LSTM performs the least competitively in terms of both error metrics and fitting ability.

From the analysis of the generalization ability of each model, the Transformer-BiLSTM model displays excellent generalization ability, as evidenced by the minimal differences between its training and testing metrics. The consistency in MAE, RMSE, and $R^{2}$ across stages suggests that it can adapt well to unseen data. Models like CNN and GRU also maintain reasonable generalization, with small gaps between their training and testing performances. This implies that these models have relatively stable performance when applied to new data. For the improved RIME-XGBoost model, although there is a noticeable difference between the training and testing metrics, its overall performance remains at a very high level, indicating strong yet slightly less consistent generalization compared to Transformer-BiLSTM. LSTM, on the other hand, exhibits the relatively weakest generalization ability among the models, with the most significant increase in error metrics from the training to the testing stage.

Through the analysis of each model architecture, ensemble learning, as exemplified by the improved RIME-XGBoost model, proves to be highly effective in achieving ultra-high prediction accuracy, making it a preferred choice when the highest precision is the primary goal. The fusion architecture of Transformer-BiLSTM combines the strengths of Transformer’s self-attention mechanism and BiLSTM’s sequential processing capability, resulting in both high accuracy and strong generalization, which is particularly suitable for tasks involving sequential data with long-range dependencies. Traditional deep learning models (GRU, CNN, and LSTM) offer decent performance but lack the breakthroughs in accuracy and generalization seen in the more advanced architectures. Among them, CNN demonstrates better performance in capturing spatial features, while GRU and LSTM handle sequential data with varying degrees of effectiveness, with GRU being relatively more efficient and stable.

In conclusion, the improved RIME-XGBoost stands out for its unparalleled prediction accuracy, Transformer-BiLSTM excels in balancing accuracy and generalization, and traditional models like CNN and GRU provide viable options for scenarios with moderate performance requirements. The choice of model should be tailored to specific task priorities, such as precision, generalization, or computational efficiency.

### 4.3. Performance Comparisons of XGBoost Based on Other Heuristic Optimization

As discussed in Section 4.2, the comprehensive predictive performance of the proposed model surpasses that of several comparable models. Section 4.3 evaluates the performance of the proposed model against other XGBoost models based on different heuristic algorithms. The goal is to investigate whether the hyperparameters optimized by improved RIME significantly outperform heuristic settings and to determine if improved RIME effectively addresses the limitations of XGBoost in hyperparameter selection. The heuristic algorithms employed in these comparative models are either those proposed in recent journal papers or widely used optimization algorithms. They include Particle Swarm Optimization (PSO) [23], Sparrow Search Algorithm (SSA) [24], Pelican Optimization Algorithm (POA) [25], Ivy Algorithm (IVY) [26], Hiking Optimization Algorithm (HOA) [27], RIME, and Improved RIME. Detailed information about them is shown in Table 5. Furthermore, regarding the parameter settings of these heuristic optimization algorithms, after parameter debugging, it was found that the number of hyperparameters is relatively small in this scenario. When the population size exceeds 10, there is no significant change in the model’s prediction accuracy. Therefore, for all the heuristic algorithms involved in the comparison, the population size is set to 20 and the maximum number of iterations is set to 50.

As shown in Table 5, PSO is renowned for its fast convergence, which enables rapid solution exploration. It is also robust, maintaining stable performance across various problem landscapes, and is easy to implement. SSA has strong global search ability and can explore a wide range of solution spaces to avoid falling into local optimization. POA-XGBoost has a simple structure, which simplifies its understanding and deployment. It can also achieve efficient optimization to ensure that the XGBoost model is effectively optimized with minimal computational overhead. IVY is flexible and can adapt well to different problem forms. It is especially good at dealing with multimodal problems, that is, problems with multiple optimal solutions, and can efficiently explore the complex solution space. HOA has fast convergence, similar to Particle Swarm Optimization (PSO), and can effectively balance exploration (searching for new areas) and development (optimizing known good areas). It can be seen that these heuristic algorithms have their own advantages, but the performance in the specific context of realizing the temperature prediction of telescopes’ back-up structure based on XGBoost still needs to be analyzed experimentally. Table 6 shows the performance results of the model optimized by these heuristic algorithms.

Comparing the performance of the models in Table 6 and Table 4, it can be seen that the XGboost model has good prediction accuracy in the thermal field of the reflector antenna’s BUS. All the heuristic algorithms selected in the experiment combined with the XGBoost model show that the performance of these models’ MAE is within 1 K, RMSE is within 1.5 K, and $R^{2}$ is more than 98%, which are significantly better than other mainstream models (such as GRU, CNN, LSTM, etc.). It can be concluded that in the specific data prediction scenario, the selection of the baseline model largely determines the final model architecture.

From the perspective of the overall prediction accuracy of the model, the performance of these XGBoost models optimized directly by heuristic algorithms is basically similar. It can be seen that the performance results of the new optimization algorithms proposed in recent years are better than those proposed in earlier years. Most importantly, the performance of the improved RIME-XGBoost model proposed in this paper is significantly better than other XGBoost models optimized directly by heuristic algorithms. The improved RIME-XGBoost also has the lowest MAE (0.331 K for the training set and 0.621 K for the test set) and RMSE (0.369 K for training set and 0.897 K for test set) in all models. In contrast, the error index of POA-XGBoost is relatively high (MAE on the test set is 1.07 K; RMSE is 1.498 K). In summary, the integration of heuristic algorithms with XGBoost yields promising predictive performance, and improved RIME-XGBoost emerges as the most robust model in terms of accuracy, generalization, and error control. The variability in performance across models underscores the importance of selecting or adapting heuristic algorithms based on the specific requirements of the prediction task.

### 4.4. Performance Verification of Improved Model Under Extreme Conditions

**Figure 9:**
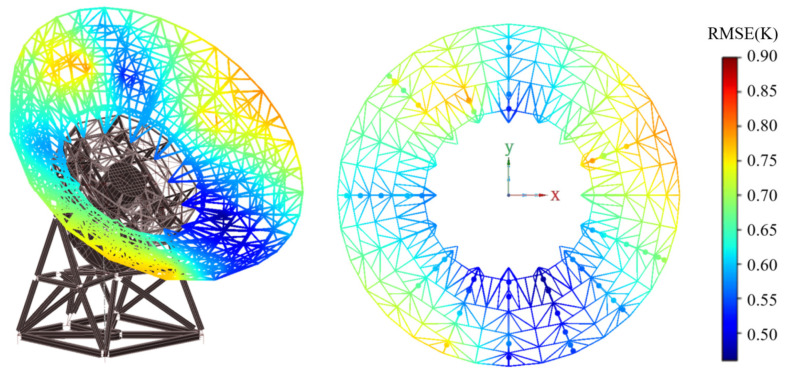
Schematic diagram of the prediction accuracy of the BUS temperature field.

Through the comparative analysis of various models and optimization algorithms, it can be concluded that the improved RIME-XGBoost model proposed in this paper has excellent performance in the scenario of obtaining the antenna’s BUS temperature. Figure 9 shows the RMSE distribution between the BUS temperature values predicted by the improved RIME-XGBoost model and the actual temperature data in the field. As shown in Figure 9, the temperature prediction accuracy in the area with more dense temperature sensors is almost within 0.7 K, and the overall prediction accuracy of the BUS temperature is within 1 K.

**Figure 10:**
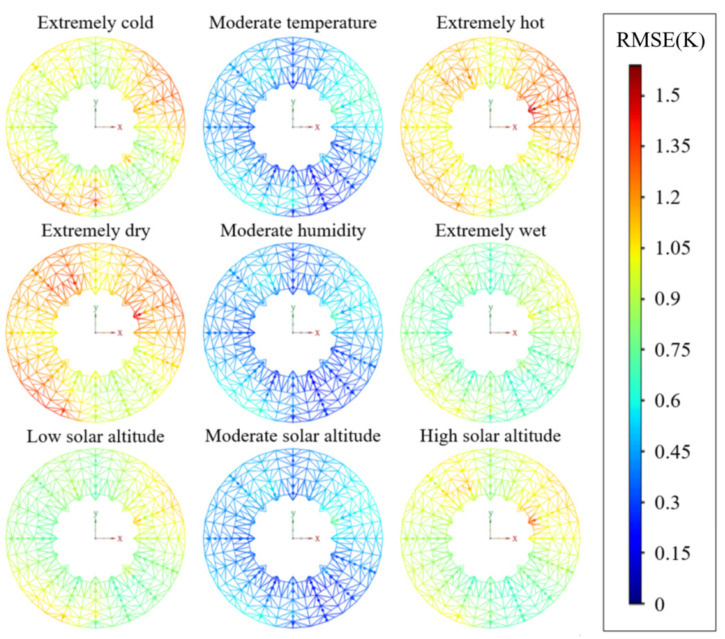
Schematic diagram of the BUS temperature field prediction accuracy under various extreme conditions.

In order to verify the performance of this model under extreme environmental conditions, we selected the three features with the strongest correlation as the key variables influencing the environmental conditions from Figure 5. These three variables are ambient temperature, relative humidity, and solar altitude. We divided these three feature variables into intervals to test the performance of the model under different environmental conditions. Table 7 represents the parameter range division information for extreme working conditions, and Table 8 presents the specific performance results of the model under each environmental condition. Figure 10 shows the visualization results of prediction accuracy.

From the analysis of the overall performance and stability of the model, the model demonstrates high predictive accuracy across most environmental conditions, as indicated by the consistently high $R^{2}$ values (all above 0.973). However, performance metrics (MAE and RMSE) exhibit variations under different extreme scenarios, suggesting that extreme environmental factors impose challenges on the model’s predictive capability.

As for the influence of ambient temperature variables on the model, when the operating condition is extremely cold or extremely hot, both temperature extremes lead to increased prediction errors (higher MAE and RMSE) and a slight decrease in $R^{2}$. Notably, extremely hot conditions affect the model more significantly than extremely cold conditions, implying that the model is more sensitive to high-temperature extremes.

For humidity conditions, compared with medium humidity, extremely dry and extremely wet environments will reduce the performance of the model. Among these, extremely dry conditions cause the most significant increase in MAE and RMSE, indicating that extreme dryness is a more critical factor for the model’s temperature prediction accuracy than extreme wetness.

As for the influence of the solar altitude angle variable on the model, the error is higher than medium solar altitude (MAE = 0.462 K, RMSE = 0.493 K, $R^{2}$ = 0.999) for both low and high solar altitudes. High solar altitude has a slightly more pronounced effect on MAE and RMSE than low solar altitude, while $R^{2}$ remains relatively high (above 0.989) under both extreme solar altitude scenarios, suggesting that the model still maintains reasonable explanatory power even under such conditions.

In summary, the model exhibits strong performance under moderate environmental conditions but experiences measurable degradation in prediction accuracy under various extreme conditions (extremely cold/hot, extremely dry/wet, and extreme solar altitudes). Among these extreme conditions, extremely dry, extremely hot, and high solar altitude conditions are the most impactful on the model’s MAE and RMSE. These findings highlight the need for further model optimization or adaptation strategies to enhance robustness in extreme environments.

### 4.5. In-Depth Discussion of Model Performance

Through a comprehensive comparative analysis of the performance data of various models across Section 4.2, Section 4.3 and Section 4.4, it can be concluded that the selection of the baseline model is crucial in the initial stage of designing the model architecture for specific prediction scenarios. An inappropriate choice is likely to result in the designed model failing to achieve the expected prediction accuracy. Additionally, if a model exhibits poor accuracy on the training set, it will inevitably perform poorly when validated on the test set. Furthermore, a model with considerable prediction accuracy on the training set does not necessarily demonstrate excellent performance on the test set. This perspective is exemplified by the model proposed in this paper: although its performance in the training set is MAE = 0.331 K, RMSE = 0.369 K, and $R^{2}$ = 0.999, its performance in the test set is MAE = 0.621 K, RMSE = 0.897 K, $R^{2}$ = 0.996 (nevertheless, its performance on the test set remains optimal compared to other models).

From the performance results, the proposed model in this paper shows an excessively rapid decline in prediction accuracy error from the training set to the test set, indicating that the generalization ability of the proposed model needs to be improved. An analysis of the model’s generalization ability under extreme operating conditions reveals that the model exhibits unsatisfactory prediction accuracy in extremely dry, extremely cold, and extremely hot environments. After analyzing the dataset, we found that this may be related to changes in the physical parameters of heat transfer involved in the structure thermal scenarios. Under extremely dry or extremely cold conditions, the convective heat transfer coefficient of the air medium in the external environment undergoes significant changes, leading to alterations in the duration of the structure temperature response. Consequently, the temperature predicted by the model at such times may suffer from temporal misalignment. Although such cases are not common, they should be the focus of future research. In addition, regarding the model’s poor performance under extremely hot conditions, an analysis of the original collected data shows that the training dataset consists of data from November to June of the following year, thus lacking summer data. This deficiency ultimately results in the model’s subpar prediction performance in extremely hot environments.

## 5. Conclusions

High precision and real-time acquisition of structure temperature data of large aperture radio telescopes is essential to ensure the long-term stable use of the antenna. This paper proposes a multi-strategy optimized RIME-XGBoost model to predict the real-time temperature of the NSRT’s BUS based on the real-time environmental conditions near the NSRT. We combine meta-heuristic optimization with ensemble learning to address the challenge of acquiring the BUS temperature data in complex outdoor environmental conditions. The key findings include the following:

(1) In the temperature prediction scenario of the reflector antenna back-up structure, the improved rime XGBoost model shows excellent prediction results. Compared with the actual temperature sensor, the prediction accuracy of the model is 97.15%, and the prediction error (RMSE) is less than 0.897 K.

(2) The XGBoost model optimized by the heuristic algorithms has good prediction accuracy in the reflector antenna back-up structure temperature prediction scenario. The performance of these models is significantly better than that of mainstream models (GRU, CNN, LSTM, and Transformer-BiLSTM), and the performance of these models includes an MAE within 1 K, RMSE within 1.5 K, and $R^{2}$ more than 98%.

(3) During the operation and service of the radio telescope, the developed model completely gets rid of the dependence on the installation and maintenance costs of physical sensors and can achieve high-precision acquisition of bus temperature by relying only on the surrounding meteorological data and the real-time mechanical parameters of the telescope, laying the foundation for the subsequent antenna thermal control and antenna health monitoring. Future work will prioritize three directions:

(1) Increase the model’s generalization ability to overcome the problem of model performance degradation in extreme environments.

(2) Explore the variation law of structure temperature and heat transfer duration and enhance the effectiveness of the dataset.

(3) Deploy a lightweight variant on the embedded system to realize the real-time thermal monitoring of the BUS.

## Figures and Tables

**Table 1:** Statistical result of the BUS temperature.

Statistical Object	Relative Humidity	Ambient Temperature	The BUS Temperature
Maximum of the average BUS temperature	22.9 RH%	293.36 K	305.655 K
Minimum of the average BUS temperature	86.5 RH%	253.45 K	260.959 K
Maximum of the point-BUS temperature	30.3 RH%	289.81 K	318.095 K
Minimum of the point-BUS temperature	85.3 RH%	255.04 K	251.511 K
Maximum of the average BUS temperature drift	36.1 RH%	−0.20 K	9.775 K/10 min
Maximum of the point-BUS temperature drift	43.4 RH%	−11.15 K	21.738 K/10 min

**Table 2:** Common chaotic mappings.

Mapping Name	Formula	Search Range
Logistic	$x_{k+1}=\lambda x_{k}\left(1-x_{k}\right),\lambda \in \left(0,4\right)$	[0, 1]
Cubic	$x_{k+1}=\rho x_{k}\left(1-x_{k}^{2}\right)$	[0, 1]
Chebyshev	$x_{k+1}=\cos\left(a\cos^{-1}\left(x_{k}\right)\right),a=0.5$	[−1, 1]
Tent	$x_{k+1}=\left\{\begin{array}{c} x_{k}/a,x_{k}<a\\ \left(1-x_{k}\right)/\left(1-a\right),x_{k}\geq a \end{array}\right.$	[0, 1]
Sine	$x_{k+1}=a\sin\left(\pi x_{k}\right),a\in \left[0,1\right]$	[0, 1]
Sinusoidal	$x_{k+1}=ax_{k}^{2}\sin\left(\pi x_{k}\right),a\in \left[0,4\right]$	[0, 1]

**Table 3:** Common models’ parameter settings.

Model	Hyperparameter
GRU	Number of neurons in GRU layer: [64, 48], the activation function: ReLU.
CNN	Number of filters in encoder part: 64, kernel parameter: 3, the activation function: ReLU.
LSTM	Number of neurons in the hidden layer: 64, the activation function: Tanh.
Transformer-BiLSTM	Number of neurons in the hidden layer of BiLSTM: 64, Head Count: 8, number of neurons in the feedforward network hidden layer: 512, the activation function: ReLU.
Improved RIME-XGBoost	Size of population: 20, number of iterations: 50

**Table 4:** Common models’ prediction performance.

Model	Stage	MAE (K)	RMSE (K)	$R^{2}$
GRU	Train	1.622	2.27	0.957
	Test	1.669	2.175	0.954
CNN	Train	1.627	2.058	0.962
	Test	1.653	2.14	0.959
LSTM	Train	1.678	2.184	0.955
	Test	1.727	2.252	0.953
Transformer-BiLSTM	Train	1.08	1.458	0.988
	Test	1.166	1.543	0.984
Improved RIME-XGBoost	Train	0.331	0.369	0.999
	Test	0.621	0.897	0.996

**Table 5:** Detailed information about comparative heuristic algorithms.

Model	Advantages of Heuristic Algorithm
PSO-XGBoost	Fast convergence, Robust, Easy to implement
SSA-XGBoost	Strong global search, High precision
POA-XGBoost	Simple structure, Efficient optimization
IVY-XGBoost	Flexible, Good at multimodal problems
HOA-XGBoost	Fast convergence, Balanced exploration and exploitation
RIME-XGBoost	Robust, Physics-inspired, Good adaptability
Improved RIME-XGBoost	Good at the temperature prediction of antenna’s BUS

**Table 6:** Heuristic models’ prediction performance.

Model	Stage	MAE (K)	RMSE (K)	$R^{2}$
PSO-XGBoost	Train	0.879	1.296	0.985
	Test	1.048	1.469	0.981
SSA-XGBoost	Train	0.76	1.179	0.989
	Test	0.945	1.359	0.985
POA-XGBoost	Train	0.911	1.355	0.985
	Test	1.07	1.498	0.982
IVY-XGBoost	Train	0.521	0.837	0.994
	Test	0.816	1.11	0.992
HOA-XGBoost	Train	0.498	0.795	0.995
	Test	0.795	1.188	0.991
RIME-XGBoost	Train	0.614	0.947	0.993
	Test	0.845	1.2	0.989
Improved RIME-XGBoost	Train	0.331	0.369	0.999
	Test	0.621	0.897	0.996

**Table 7:** Information on the parameter range division for extreme working conditions.

Working Conditions	Interval Division Explanation
Extremely cold	The environmental temperature is below 258.15 K.
Moderate temperature	The environmental temperature ranges from 283.15 K to 293.15 K.
Extremely hot	The environmental temperature is above 293.15 K.
Extremely dry	The environmental humidity is below 15%.
Moderate humidity	The environmental humidity ranges from 35% to 60%.
Extremely wet	The environmental humidity is above 80%.
Low solar altitude	The solar altitude angle is below 20 degrees.
Moderate solar altitude	The solar altitude angle ranges from 30 degrees to 45 degrees.
High solar altitude	The solar altitude angle is above 55 degrees.

**Table 8:** The performance of the model under extreme conditions.

Environmental Conditions	MAE (K)	RMSE (K)	$R^{2}$
Extremely cold	0.849	1.281	0.984
Moderate temperature	0.564	0.544	0.998
Extremely hot	0.937	1.345	0.975
Extremely dry	0.954	1.396	0.973
Moderate humidity	0.529	0.554	0.997
Extremely wet	0.817	1.014	0.991
Low solar altitude	0.627	1.099	0.995
Moderate solar altitude	0.462	0.493	0.999
High solar altitude	0.746	1.141	0.989

## Data Availability

The data presented in this study are available on request from the corresponding author.

## Abbreviations

The following abbreviations are used in this manuscript:

BUS	back-up structure
XGBoost	eXtreme Gradient Boosting
NSRT	Nanshan 26-m Radio Telescope
RMSE	root mean square error
IRAM	Institute of Radioastronomie Millimétrique
SRT	Sardinia Radio Telescope
GBT	Green Bank Telescope
FBG	Fiber Bragg Grating
PTFE	polytetrafluoroethylene
OBL	opposition-based learning
ROBL	refractive opposition-based learning
MSE	mean squared error
MAE	mean absolute error
ASTE	Atacama Submillimeter Telescope Experiment
OOF	Out of Focus
FEM	finite element method
CNN	convolutional neural network
LSTM	long short-term memory
BARO	barometric pressure
GRU	Gated Recurrent Unit
